# The co-evolution of social networks and health-related behaviors in university students: A two-year longitudinal network study

**DOI:** 10.1016/j.ijchp.2026.100720

**Published:** 2026-09-09

**Authors:** Rui Luo, Yongqiang Li, Jingyu Zhang, Jinming Liu, Chun Hao, Jinghua Li, Jing Gu

**Affiliations:** a Department of medical statistics, School of Public Health, Sun Yat-Sen University, Guangzhou, China; b Arnold School of Public Health, University of South Carolina, 915 Greene St., Columbia, SC, US; c Department of Maternal and Child Health, School of Public Health, Sun Yat-sen University, Guangzhou, China; d Department of Public Health and Medicinal Administration, Faculty of Health Sciences, University of Macau, Avenida da Universidade, Taipa, Macau, China; e Sun Yat-sen University Global Health Institute, School of Public Health and Institute of State Governance, Sun Yat-sen University, Guangzhou, 510080, China; f Guangdong Key Laboratory of Health Informatics, Guangzhou, 510080, China

**Keywords:** Social network analysis, Health-related behaviors, Stochastic actor-oriented model (SAOM), Contagion and selection, Longitudinal study, University students

## Abstract

Health-related behaviors adopted during emerging adulthood have important implications for long-term health trajectories. While homophily has been widely documented for substance use, less is known about whether behavioral similarity arises from contagion or selection across a broader range of health-related behaviors. Distinguishing between these mechanisms has direct implications for intervention design. We conducted a four-wave longitudinal sociocentric cohort study of undergraduate freshmen at two universities in Guangzhou, China (October 2022–July 2024). Eligible participants from five schools completed online surveys at baseline and three follow-up waves. Measures included physical activity, fruit and vegetable consumption, sleep quality, smoking, drinking, sugar-sweetened beverage (SSB) consumption, and video gaming. Friendship networks were assessed using roster-based nominations. Stochastic Actor-Oriented Models were used to examine the co-evolution of networks and behaviors and to estimate the relative contributions of contagion and selection processes. Parameter estimates were pooled across schools using meta-analytic procedures. The analytic sample included 609 students at baseline (mean age = 18.7 years; 65.2% female). Significant contagion effects were observed for physical activity, fruit consumption, sleep quality, smoking, drinking, and video gaming. In contrast, homophily in SSB consumption was primarily explained by selection processes. Overall, contagion accounted for a larger share of observed homophily than selection, with stronger effects for health-risk behaviors than for health-promoting behaviors. Mechanisms generating peer similarity vary by behavior. These findings suggest that mechanism-informed interventions, network diffusion strategies for influence-sensitive behaviors and structural or individual-level approaches for selection-driven behaviors, may help inform intervention design during the transition to university, although this study did not test network-based interventions.

## Introduction

Health-related behaviors established during adolescence and emerging adulthood have profound implications for health trajectories across the life course (Larson, 1990). A substantial proportion of premature morbidity and mortality in adulthood can be traced to modifiable behavioral risk factors adopted during these developmental periods (World Health Organization, 2009). Globally, the prevalence of unhealthy behaviors among young people remains high: insufficient physical activity, inadequate fruit and vegetable consumption, drinking and smoking, sleep disturbances, and excessive screen time are widely documented across diverse contexts (Guedes & Silva, 2021; Guthold et al., 2020; Jiang et al., 2015; World Health Organization, 2024). Given the rapid biological, psychological, and social transitions that characterize adolescence and emerging adulthood, understanding how health-related behaviors are shaped and sustained during this stage is a critical public health priority.

Health-related behaviors do not develop in isolation. Adolescents and young adults are embedded within social networks that structure opportunities, norms, and exposures. A consistent finding in social network research is behavioral homophily, the tendency for individuals with similar behaviors to cluster within networks (McPherson et al., 2001). Homophily in smoking, drinking, and other risk behaviors has been widely documented (Christakis & Fowler, 2008; Daw et al., 2015; Farhat et al., 2012). One explanation for this clustering is social contagion, whereby individuals adopt behaviors through peer influence and social reinforcement (Steglich et al., 2010). Through mechanisms such as imitation, shared norms, and social learning, behaviors, both health-promoting and health-compromising, may diffuse across networks.

However, observed behavioral similarity may also arise from selection processes, whereby individuals preferentially form or maintain ties with others who already share similar characteristics or behaviors (Kandel, 2017). The coexistence of contagion and selection poses a longstanding methodological challenge in network research. Disentangling these mechanisms is crucial because they imply fundamentally different intervention strategies: if contagion dominates, altering key actors or network structures may shift behaviors; if selection prevails, interventions may need to target individual predispositions before network clustering occurs.

Stochastic Actor-Oriented Modeling (SAOM) provides a rigorous framework for distinguishing between selection and contagion processes in longitudinal social networks (Snijders et al., 2010). By modeling the co-evolution of networks and behaviors, SAOM enables researchers to estimate the relative contribution of contagion and selection mechanisms. Existing SAOM-based studies of adolescent health behaviors have largely focused on substance use, particularly smoking, drinking, and marijuana use, with mixed empirical findings (Barnett et al., 2022; Haas & Schaefer, 2014; Ivaniushina & Titkova, 2021; Mercken et al., 2010; Osgood et al., 2013). Moreover, much of this evidence derives from the Add Health cohort in the United States. While foundational, this dataset reflects cohorts recruited decades ago and may not capture contemporary social environments shaped by digital communication, shifting cultural norms, and changing educational contexts. Additionally, cross-cultural differences may influence both network structures and behavioral diffusion processes. Importantly, far less attention has been paid to the contagion dynamics of health-promoting behaviors, such as physical activity, healthy diet, and sleep patterns.

University students represent a particularly salient population for examining these processes. Positioned at the intersection of late adolescence and emerging adulthood, university students experience expanded autonomy, restructured peer networks, and heightened exposure to both risky and health-promoting behaviors (Arnett, 2000; Miller et al., 2002; Storer et al., 1997). During this transitional period, peer networks may play a pivotal role in reinforcing or reshaping behavioral norms. Yet the relative contributions of selection and contagion mechanisms across multiple domains of health-related behavior remain insufficiently understood in contemporary university contexts. The Chinese university setting provides a well-bounded context for studying peer processes. Unlike systems where undergraduates freely mix across cohorts and institutions, Chinese universities assign entering students to a fixed major and class, in which they take most coursework together, are commonly housed together, and remain throughout their program. First-year students' friendships and daily co-activities are thus concentrated within the same-cohort, same-school environment, the dominant arena of peer influence during this transition, making the within-cohort network a meaningful boundary for examining selection and contagion.

To address these gaps, the present study has three objectives: (1) to examine the extent of behavioral homophily across multiple health-related behaviors among university students; (2) to disentangle the roles of selection and contagion mechanisms underlying observed homophily; and (3) to quantify the relative contribution of these mechanisms for each behavior using longitudinal network modeling. By clarifying how different health behaviors co-evolve with social networks, this study contributes to the growing literature on social determinants of health within peer contexts and provides evidence to inform network-based public health interventions in university settings.

## Methods

### Participants

**Fig. 1:**
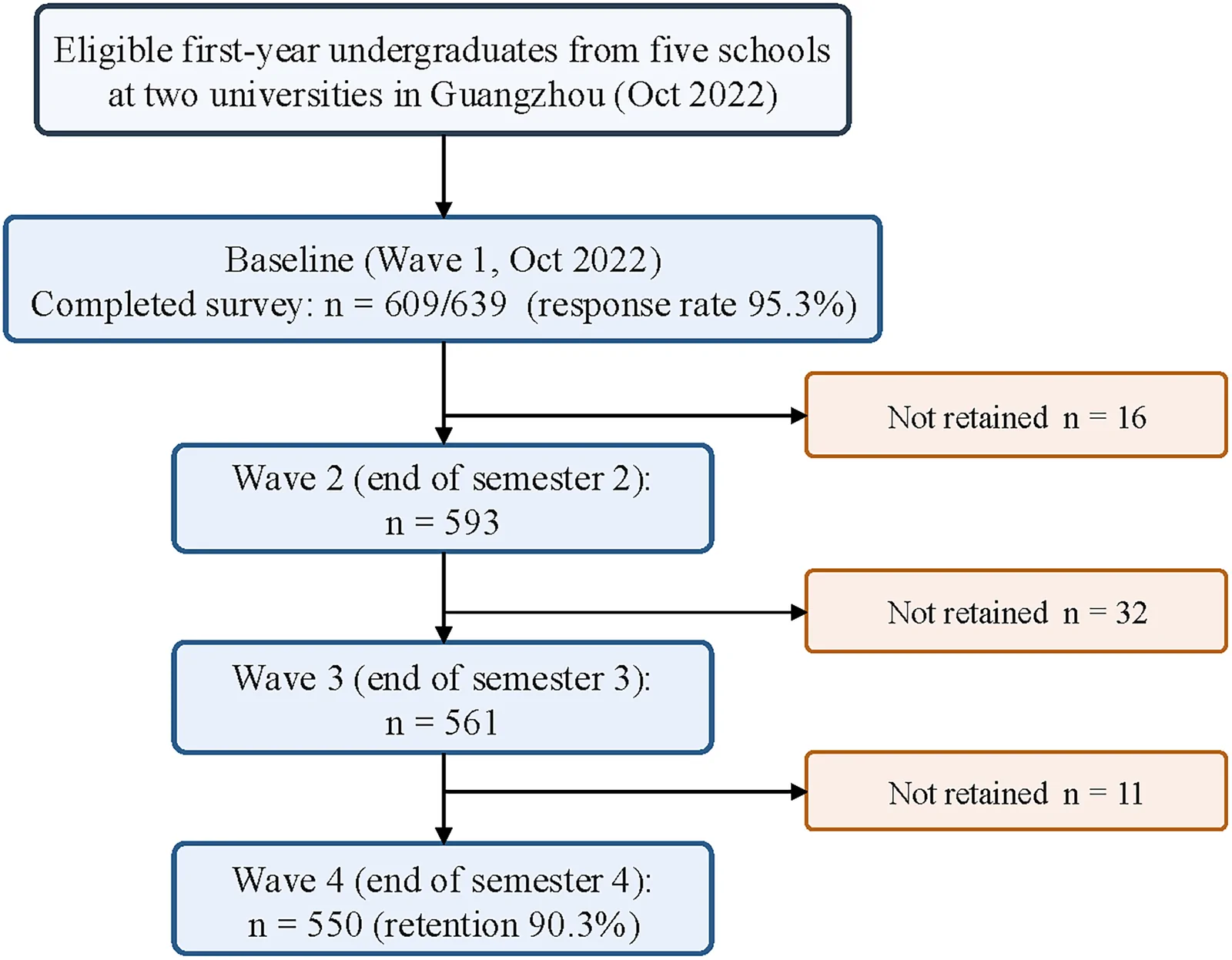
Flowchart of participant recruitment and follow-up across the four waves.

A cohort study was conducted at two universities in Guangzhou, Guangdong Province, from October 2022 to July 2024, with data collected in four waves. The study employed sociocentric network design, encompassing the entire network of undergraduate freshmen. Purposive sampling was used to recruit eligible freshmen from five selected schools (including medical sciences, liberal arts, and engineering) within the two universities. Inclusion criteria were: (1) enrolled as an undergraduate freshman in September 2022 and (2) affiliated with one of the selected schools. Exclusion criteria were: (1) not fully registered; (2) refusal to participate; (3) inability to participate due to severe physical or mental illness. At baseline (Wave 1), 609 students completed the survey. Follow-up participation was 593 (Wave 2), 561 (Wave 3), and 550 (Wave 4), corresponding to a retention rate of 90.3% at the final wave. The participant flow, including the numbers eligible, enrolled at baseline, retained at each wave, and included in the final analysis, is shown in Fig. 1.

### Study design and procedures

We introduced the study to undergraduate students through classroom presentations, during which we explained the study’s background, objectives, procedures, potential benefits, and risks. After the presentations, students had the option to schedule appointments online, allowing them to visit the designated survey site at a convenient time. The survey site was in the university’s student activity center. Upon arrival, study assistants verified each student’s eligibility and obtained informed consent. Eligible participants were then invited to complete the online baseline assessment. The follow-up assessments were conducted at the end of the second, third, and fourth academic semesters, respectively. All assessments were completed anonymously using the Questionnaire Star online platform (www.wjx.cn), with each survey taking approximately 15 min to complete.

### Measurements

#### Physical exercise

Physical exercise level was measured using the revised Physical Activity Rank Scale-3 (PARS-3) by Liang et al. (Liang, 1994). The scale consists of three items, utilizing a Likert-type 5-point scoring system to assess the intensity, duration, and frequency of engaging in Physical activity. The scoring method is as follows: Physical Activity Score = Intensity × (Duration - 1) × Frequency, with a maximum score of 100 and a minimum of 0. The assessment criteria for physical activity levels were low intensity ≤19 points, moderate intensity 20-42 points, high intensity ≥43 points. The scale demonstrates high reliability and validity.

#### Fruit and vegetable consumption

The measurement of fruit and vegetable consumption was similar to the questions in the Food frequency questionnaire (FFQ) (Cade et al., 2002). Surveyors assessed the amount of fruit and vegetable consumption by asking participants about the frequency of eating fruits (excluding fruit juice) and the frequency of consuming vegetables or salads (excluding starchy foods like potatoes). Nine frequency response options were provided, ranging from never to two or more times per day (never; once/month; 2–3 times/month; once/week; twice/week; 3–4 times/week; 5–6 times/week; once/day; ≥ twice/day), with higher scores indicating higher intake.

#### Sleep quality

The Pittsburgh Sleep Quality Index (PSQI), comprising 19 items, was utilized to assess sleep quality (Buysse et al., 1989). The PSQI consists of 7 dimensions, with each dimension scored on a scale of 0-3 points. The total PSQI score ranges from 0 to 21, with higher scores indicating poorer sleep quality. A score of 0–5 is considered indicative of good sleep quality, while a score above 5 suggests poor sleep. In our study, the Cronbach's α of the PSQI was 0.862.

#### Smoking

The smoking frequency of the participants in the past month was determined using the smoking section of the Tobacco Survey Questionnaire developed by the World Health Organization (WHO) (World Health Organization & Centers for Disease Control and Prevention, 2019). This was specifically assessed through the following question: "In the past month, on how many days did you smoke?" (referring to machine-rolled or hand-rolled cigarettes). Response options included never, 1–2 days, 3–5 days, 6–9 days, 10–19 days, 20–29 days, and every day, comprising seven categories; adjacent categories were collapsed for presentation in Table 3.

#### Drinking

Alcohol consumption was assessed using the past-month drinking-frequency item (the first item) of the Alcohol Use Disorders Identification Test (AUDIT) (Saunders et al., 1993); this frequency item is the measure used in the analyses and reported in Table 3. For instance, the question is posed as follows: "In the last month, how often did you consume alcoholic beverages?" The response options include never, once a month, 2-4 times a month, 2-3 times a week, and ≥4 times a week. This scale demonstrates good reliability and validity.

#### Consumption of sugar sweetened beverages

The consumption of sugar sweetened beverages in the past month was assessed using the Diet History Questionnaire III (DHQ III) (National Cancer Institute, 2024). This study included eight types of sugary beverages commonly found in China, such as sweetened tea drinks, sweetened coffee drinks, sweetened dairy beverages, and sweetened carbonated drinks. For each participant, SSB consumption was defined as the highest reported frequency across the eight beverage types (a row-wise maximum), then collapsed into seven ordered categories; higher values indicate more frequent consumption of the most-frequently-consumed beverage.

#### Video game playing

To evaluate the average time spent per week on electronic gaming over the past month (in hours), participants were asked two questions: 'Approximately how many hours per day did you spend playing games in the last month? ' and 'In the last month, how many days per week did you usually play games?' Responses were then categorized into five frequency options, ranging from 'Never' to 'More than 14 h per week.

#### Background characteristics

Sociodemographic information was collected through a questionnaire, including sex, age, monthly allowance, household registration, parental educational background, only-child status, height, and weight.

#### Social networks

Sociocentric social network data were collected using the Fixed List Selection method (Doreian & Woodard, 1992). Participants were asked to select the names of 10 classmates from their grade in an electronic questionnaire. The questionnaire preloaded the list of undergraduate freshmen from their respective colleges, serving as the boundary for our sociocentric social network, to facilitate participants in choosing their friendship relationships. Subsequently, participants were asked to indicate the intimacy level with each selected friend ("casual," "close," "intimate"). Friendship ties were dichotomized into binary directed networks: a directed tie from i to j was coded as present if i nominated j and absent otherwise, regardless of the reported intimacy level. Intimacy ratings were collected to describe tie composition and were not used to weight ties, because the stochastic actor-oriented co-evolution models and diagnostics used here are formulated for binary directed networks (Ripley et al., 2011; Snijders et al., 2010). Directed adjacency matrices were constructed for each wave, and network dynamics were analyzed separately within each school to preserve complete network structure.

### Statistical analysis

We performed descriptive statistics, calculating frequencies and percentages for categorical variables, and means and standard deviations for continuous variables. Differences across the four waves were tested with the Friedman test for ordinal behaviors and the McNemar test for the binary sleep-quality variable. Linear trends in homophily across the four waves were assessed using a test for linear trend across waves. Behavioral homophily was quantified with the Association Coefficient, the network assortativity coefficient for a behavioral attribute (Yuan et al., 2021), which measures the tendency of connected actors to share similar behavior levels and ranges from −1 (disassortative) to 1 (assortative), with values near 0 indicating no association.

The analyses used SAOM to discern contagion and selection (Steglich et al., 2010). Contagion was set to the behaviors average alter parameter (Ripley et al., 2011). In addition, in line with prior studies (Snijders, 2001), we included key characteristics of the current network, such as reciprocity, transitive triplets, and background characteristics, as covariates. The final coding of all eight behavioral variables for the SAOM behavioral submodel is summarized in Table S2. Continuous or wide-range scales (physical activity, PSQI, and SSB frequency) were collapsed into ordered categories as required by the model, and sleep quality was reverse-coded so that higher values denote better sleep, ensuring that all behavioral coefficients are interpreted in a consistent direction.

Each behavior was modeled separately in its own network–behavior co-evolution model, consistent with standard SAOM practice. To account for multiple comparisons across the eight behavioral models, contagion (average-alter) *p*-values were adjusted using the Benjamini-Hochberg false discovery rate procedure. Because friendship networks were bounded within each school, the five networks were structurally separate and were not merged. Because the online survey required responses to all items, there was no item-level missingness; the only missingness was panel attrition. Given the high retention (90.3%), the SAOM analyses were restricted to the 550 participants with complete data at all four waves (complete-case analysis). A separate model with an identical specification was estimated for each of the five schools in RSiena using the method of moments. Convergence was evaluated with the overall maximum convergence ratio (< 0.25) and per-parameter convergence t-ratios (|t| < 0.10); models not meeting these criteria were re-estimated with additional subphases (Table S4).

School-specific estimates were then combined using the random-effects meta-analytic procedure of Snijders and Baerveldt (2003), which estimates the mean of each effect across schools using inverse-variance weighting while allowing for between-school variation. As each model was estimated within a single school, university- and school-level contextual differences were absorbed by design rather than modeled with dummy variables. The full model specification, network effects (outdegree, reciprocity, transitive triplets, 3-cycles, degree-related and covariate effects) and behavior effects (linear and quadratic shape, average-alter, degree-related, and covariate effects), is reported in Table S1. Model convergence was assessed using the overall maximum convergence ratio and per-parameter t-ratios, and goodness of fit was evaluated with sienaGOF for the indegree, outdegree, and behavior distributions. Representative GOF plots are shown in Figs. S1–S2. Time heterogeneity across the three observation periods was assessed with the score-type test of Lospinoso et al. (2011) (implemented as sienaTimeTest in RSiena); the joint test was non-significant for all models (all *p* > 0.05; Table S6).

**Fig. 2:**
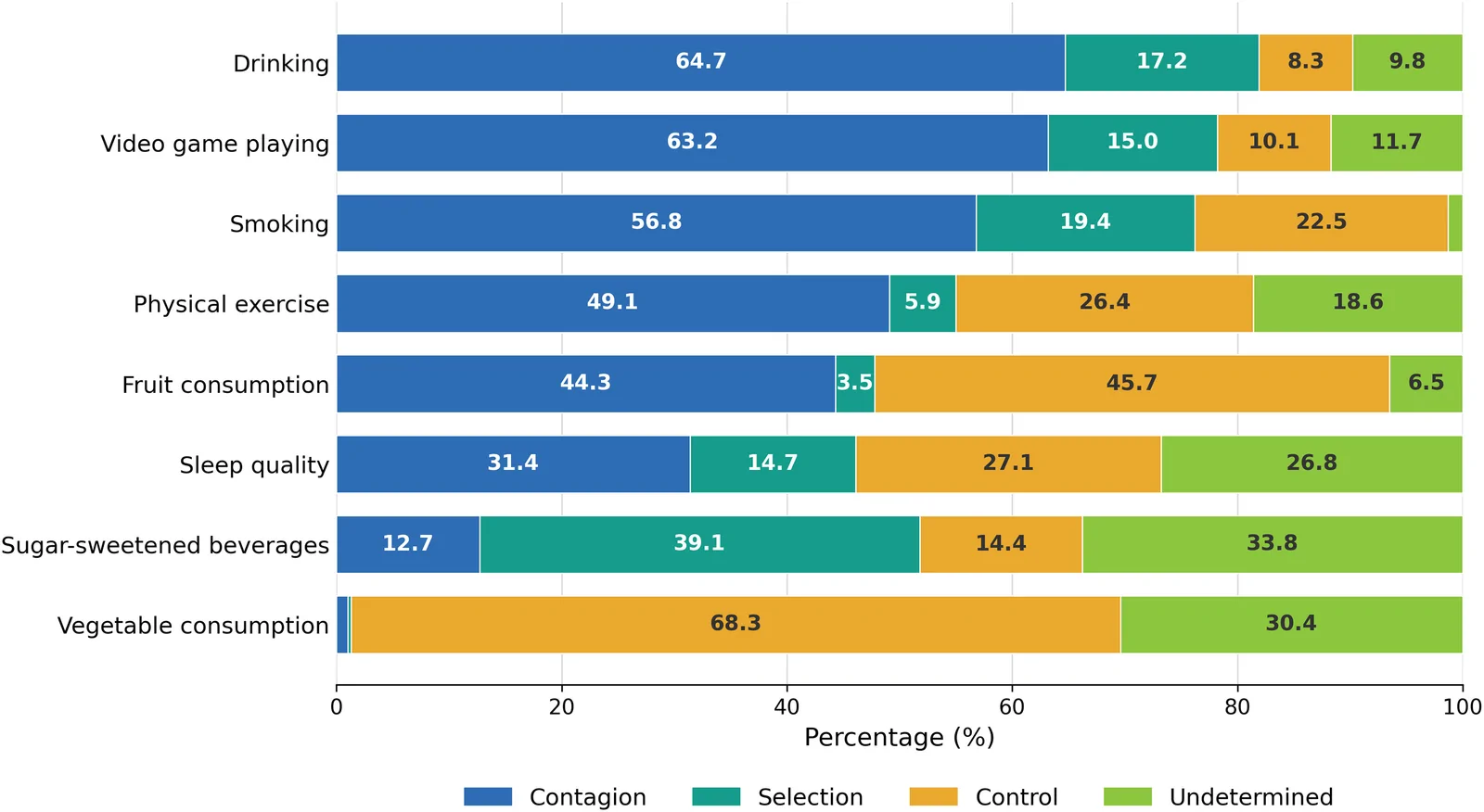
Mechanisms underlying changes in health behaviors.

The relative contributions of four mechanisms to the observed behavioral homophily (Fig. 2) were estimated using the simulation-based decomposition of Steglich et al. (2010). From each fitted model, we simulated the co-evolution of the network and behavior (1000 runs) and quantified each mechanism's contribution as the change in simulated behavioral assortativity when its parameter(s) were constrained to zero relative to the full model, expressed as a percentage (components summing to 100%). The contagion component reflects the average-alter effect; the selection component reflects the behavior similarity effects; the control component reflects structural network effects (reciprocity, transitivity, degree) and background-covariate homophily that generate similarity through mechanisms other than selection or influence; and the undetermined component is the residual not uniquely attributable to a single mechanism. Statistical significance was set at *p* < 0.05. All analyses were performed using R version 4.3.1.

## Results

### Participant characteristics and network stability

A total of 609 freshmen were included at baseline, of whom 65.2% were female and 29.7% originated from rural areas. Approximately 44% had at least one parent with college education or above, and 23.1% were classified as overweight or obese. Detailed characteristics are presented in Table 1. Across four waves, network density remained stable at 0.01, while the total number of friendship ties declined from 2756 at Wave 1 to 2264 at Wave 4. Reciprocity increased slightly over time (0.75 to 0.79), indicating growing mutuality in friendship nominations. All Jaccard indices exceeded 0.30 (0.74, 0.75, and 0.79), satisfying the temporal stability assumption required for SAOM estimation. Details are provided in Table 2.

**Table 1:** Background characteristics at baseline.

Variables	School 1 (N=82)	School 2 (N=91)	School 3 (N=81)	School 4 (N=255)	School 5 (N=100)	All (N=609)
Sex						
Male	37 (45.1)	46 (50.5)	22 (27.2)	48 (18.8)	59 (59.0)	212 (34.8)
Female	45 (54.9)	45 (49.5)	59 (72.8)	207 (81.2)	41 (41.0)	397 (65.2)
Age at wave 1 (mean ± sd)	18.7±0.6	18.9±0.5	18.4±0.8	18.8±0.6	18.2±0.7	18.7±0.6
Only-child status						
Yes	32 (39.0)	31 (34.1)	36 (44.4)	53 (20.8)	45 (45.0)	197 (32.3)
No	50 (61.0)	60 (65.9)	45 (55.6)	202 (79.2)	55 (55.0)	412 (67.7)
Monthly allowance (yuan)						
<1000 (138 USD)	3 (3.7)	10 (11.0)	1 (1.3)	35 (13.7)	6 (6.0)	55 (9.0)
1000-2000 (138-276 USD)	42 (51.1)	46 (50.5)	33 (40.7)	178 (69.8)	42 (42.0)	341 (56.0)
2001-3000 (277-414 USD)	34 (41.5)	24 (26.4)	33 (40.7)	36 (14.1)	38 (38.0)	165 (27.1)
>3000 (414 USD)	3 (3.7)	11 (12.1)	14 (17.3)	6 (2.4)	14 (14.0)	48 (7.9)
Household registration						
Urban	61 (74.4)	74 (81.3)	68 (84.0)	140 (54.9)	85 (85.0)	428 (70.3)
Rural	21 (25.6)	17 (18.7)	13 (16.0)	115 (45.1)	15 (15.0)	181 (29.7)
Parental educational background*						
Junior high school or below	17 (20.7)	15 (16.5)	13 (16.0)	122 (47.8)	13 (13.0)	180 (29.6)
High school or technical secondary school	18 (22.0)	30 (33.0)	25 (30.9)	71 (27.8)	17 (17.0)	161 (26.4)
College or above	47 (57.3)	46 (50.5)	43 (53.1)	62 (24.3)	70 (70.0)	268 (44.0)
BMI (kg·m^−2^)						
Underweight (<18.5)	13 (15.9)	18 (19.8)	12 (14.8)	77 (30.2)	20 (20.0)	140 (23.0)
Normal (18.5∼22.9)	46 (56.1)	51 (56.0)	51 (63.0)	123 (48.2)	57 (57.0)	328 (53.9)
Overweight (23∼24.9)	15 (18.3)	15 (16.5)	11 (13.6)	24 (9.4)	15 (15.0)	80 (13.1)
Obesity (≥25)	8 (9.7)	7 (7.7)	7 (8.6)	31 (12.2)	8 (8.0)	61 (10.0)

⁎ : the educational background of the father or mother with a higher education level in the family takes precedence.

**Table 2:** network-related indicators over waves.

Variables	Wave 1 (n=609)	Wave 2 (n=593)			Wave 3 (n=561)			Wave 4 (n=550)
Network density indicators	0.01	0.01			0.01			0.01
Number of edges	2756	2553			2342			2264
Weight of edges								
Casual friendship	1188 (43.1)	866 (33.9)			714 (30.5)			640 (28.3)
Close friendship	1314 (47.7)	1326 (51.9)			1375 (58.7)			1363 (60.2)
Intimate friendship	254 (9.2)	361 (14.1)			253 (10.8)			261 (11.5)
Mean of indegree	4.53	4.31			4.17			4.12
Reciprocity index	0.75	0.74			0.77			0.79
Sample change	Period1 (wave1-wave2)			Period2 (wave2-wave3)			Period3 (wave3-wave4)	
Number of ties dissolved	342			298			235	
Number of ties emerged	405			376			302	
Number of ties maintained	2148			1966			1962	
Jaccard index *	0.74			0.75			0.79	

⁎ : The Jaccard index quantifies the degree of change between observed waves, indicating whether the data collection points are sufficiently close in time. Values greater than 0.3 are preferred to ensure that the network change process is gradual is met. Tie dissolution, formation, and maintenance (and the Jaccard index) were computed among actors present in both adjacent waves; the number of edges refers to all actors present in each wave.

### Changes in health-related behaviors over time

Several behavioral trajectories shifted during the two-year follow-up. The proportion of students reporting no physical exercise decreased modestly (20.8% to 16.2%, *p*=0.021), and low-frequency fruit and vegetable consumption declined. In contrast, sleep quality worsened, with good sleep decreasing from 60.6% to 47.5% (*p*=0.013). Risk behaviors showed increasing prevalence: the proportion of students reporting any drinking increased over time, and the percentage of students who never engaged in video gaming declined substantially (50.8% to 39.6%, *p*<0.001). These findings suggest a mixed behavioral pattern during the transition into university life. Details are provided in Table 3.

**Table 3:** Health-related behaviors over waves.

Variables	Wave 1 (n=609)	Wave 2 (n=593)	Wave 3 (n=561)	Wave 4 (n=550)	*p*
Physical exercise					
Never	127 (20.8)	92 (15.5)	105 (18.9)	89 (16.2)	0.021^a^
Low intensity	320 (52.5)	319 (53.8)	287 (51.1)	290 (52.8)	
Moderate intensity	96 (15.7)	106 (17.9)	95 (16.9)	97 (17.7)	
High intensity	66 (10.9)	76 (12.8)	74 (13.1)	74 (13.3)	
Vegetable consumption					
≤ once/week	55 (9.0)	47 (8.0)	40 (7.1)	27 (4.9)	0.004^a^
2-4 times/week	149 (24.5)	185 (31.2)	190 (33.9)	179 (32.5)	
5-6 times/week	73 (12.0)	96 (16.2)	71 (12.7)	67 (12.2)	
Once/day	178 (29.2)	141 (23.8)	147 (26.2)	165 (30.0)	
≥2 times/day	154 (25.3)	124 (20.8)	113 (20.1)	112 (20.4)	
Fruit consumption					
≤ once/week	209 (34.2)	159 (26.8)	177 (31.6)	158 (28.7)	0.005^a^
2-4 times/week	275 (45.1)	279 (47.0)	271 (48.3)	263 (47.9)	
5-6 times/week	44 (7.3)	57 (9.6)	39 (6.9)	47 (8.6)	
Once/day	55 (9.1)	54 (9.2)	52 (9.3)	54 (9.9)	
≥2 times/day	26 (4.3)	44 (7.4)	22 (3.9)	28 (4.9)	
Sleep quality					
Good	369 (60.6)	301 (50.8)	281 (50.1)	261 (47.5)	0.013^b^
Poor	240 (39.4)	292 (49.2)	280 (49.9)	289 (52.5)	
Smoking					
Never	598(98.2)	578 (97.5)	541 (96.4)	528 (96.0)	0.023^a^
1-5 days /month	4 (0.6)	7 (1.1)	8 (1.4)	11 (2.0)	
6-19days /month	3 (0.5)	4 (0.7)	5 (0.9)	3 (0.5)	
20-29days /month	1 (0.2)	0 (0.0)	1 (0.2)	1 (0.2)	
Every day	3 (0.5)	4 (0.7)	6 (1.1)	7 (1.3)	
Drinking					
Never	435 (71.5)	422 (71.1)	417 (74.3)	356 (64.8)	<0.001^a^
Once/ month	99 (16.4)	109 (18.4)	80 (14.3)	113 (20.6)	
2-4 times/ month	61 (10.0)	50 (8.4)	52 (9.3)	69 (12.5)	
2-3 times/ week	14 (2.2)	9 (1.6)	8 (1.4)	9 (1.7)	
≥ 4 times/ week	0 (0.0)	3 (0.5)	4 (0.7)	3 (0.5)	
Consumption of sugar sweetened beverages					
Never	15 (2.5)	15 (2.5)	17 (3.0)	18 (3.2)	<0.001^a^
<Once/ week	104 (17.1)	86 (14.5)	114 (20.3)	124 (22.5)	
1-2 times/ week	198 (32.5)	204 (34.4)	217 (38.7)	190 (34.5)	
3-4 times/ week	145 (23.8)	143 (24.1)	107 (19.1)	105 (19.1)	
5-6 times/ week	72 (11.8)	70 (11.8)	57 (10.2)	47 (8.5)	
Once/ day	47 (7.7)	36 (6.1)	27 (4.8)	39 (7.1)	
≥2 times/ day	28 (4.6)	39 (6.6)	22 (3.9)	27 (4.9)	
Video game playing					
Never	309 (50.8)	276 (46.5)	272 (48.2)	218 (39.6)	<0.001^a^
≤3 h/ week	66 (10.9)	73 (12.3)	60 (10.7)	80 (14.6)	
3-7 h/ week	90 (14.8)	88 (14.8)	87 (15.5)	107 (19.4)	
7-14 h/ week	64 (10.5)	76 (12.8)	67 (12)	82 (14.9)	
>14 h/ week	80 (13.0)	80 (13.5)	75 (13.6)	63 (11.5)	

a : The Friedman test was used to examine differences among the 550 participants who had data at all time points.

b : The McNemar test was used to examine differences among the 550 participants who had data at all time points.

### Behavioral homophily within friendship networks

Among health-promoting behaviors, homophily in physical exercise was statistically significant across all four waves, whereas homophily in fruit consumption reached significance from Wave 2 to Wave 4. Specifically, the assortativity coefficients for physical activity were 0.120 (*p* < 0.001), 0.133 (*p* < 0.001), 0.151 (*p* < 0.001), and 0.176 (*p* < 0.001) from Wave 1 to Wave 4, respectively. For health-risk behaviors, homophily in smoking and drinking also reached statistical significance at all four waves. The assortativity coefficients for drinking were 0.057 (*p* = 0.013), 0.086 (*p* < 0.001), 0.151 (*p* < 0.001), and 0.187 (*p* < 0.001), respectively. Moreover, except for vegetable consumption and sleep, most health-related behaviors exhibited a consistent upward trend in homophily over time. Detailed results are presented in Table 4.

**Table 4:** homophily and trends of different health-related behaviors.

Variables	Association Coefficient	SE	*p*	*p*_trend_
Physical exercise				
Wave 1	0.120	0.022	<0.001	0.011
Wave 2	0.133	0.024	<0.001	
Wave 3	0.151	0.022	< 0.001	
Wave 4	0.176	0.026	<0.001	
Vegetable consumption				
Wave 1	0.050	0.022	0.023	0.900
Wave 2	0.015	0.023	0.514	
Wave 3	0.064	0.022	0.002	
Wave 4	0.028	0.026	0.282	
Fruit consumption				
Wave 1	0.036	0.023	0.118	0.048
Wave 2	0.046	0.024	0.048	
Wave 3	0.063	0.021	0.002	
Wave 4	0.104	0.026	< 0.001	
Sleep quality				
Wave 1	0.013	0.022	0.555	0.191
Wave 2	-0.019	0.024	0.429	
Wave 3	0.046	0.022	0.037	
Wave 4	0.082	0.026	0.003	
Smoking				
Wave 1	0.048	0.020	0.016	0.043
Wave 2	0.111	0.022	0.003	
Wave 3	0.116	0.021	0.002	
Wave 4	0.141	0.025	< 0.001	
Drinking				
Wave 1	0.057	0.023	0.013	0.005
Wave 2	0.086	0.024	<0.001	
Wave 3	0.151	0.022	<0.001	
Wave 4	0.187	0.026	<0.001	
Consumption of sugar sweetened beverages				
Wave 1	0.012	0.022	0.585	0.025
Wave 2	0.026	0.023	0.258	
Wave 3	0.038	0.022	0.084	
Wave 4	0.068	0.026	0.006	
Video game playing				
Wave 1	0.007	0.022	0.750	0.018
Wave 2	0.107	0.024	<0.001	
Wave 3	0.137	0.024	<0.001	
Wave 4	0.217	0.023	<0.001	

### Disentangling contagion and selection processes

Table 5 presented the results of SAOM describing the coevolution of friendship networks and the studied behaviors. All school-specific and behavior-specific models converged satisfactorily (overall maximum convergence ratio < 0.25; all |t| < 0.10). Goodness-of-fit checks indicated adequate fit for the large majority of models: of 120 distribution-by-model comparisons, 116 were non-significant (*p* > 0.05) and only four fell marginally below 0.05 (complete p-values in Table S5; representative plots in Figs. S1–S2). Significant positive contagion effects were identified in physical exercise (*b* =1.591, *p*<0.001), fruit consumption (*b* =1.467, *p*<0.001), sleep quality (*b* =1.143, *p*<0.001), smoking (*b* =1.851, *p*=0.003), drinking (*b* =2.232, *p*<0.001) and video game playing (*b* =2.356, *p*<0.001). The contagion effects of vegetable consumption and consumption of sugar sweetened beverages were not significant. The results indicated that only the selection effect of sugar sweetened beverages consumption was significant (*b* =1.352, *p*<0.001), while the selection effects of other health-related behaviors were not significant. The results of other control variables were detailed in Table S3. All six significant contagion effects remained significant after Benjamini-Hochberg FDR correction (all *q* < 0.01).

**Table 5:** SAOM estimates for friendship network and health-related behaviors dynamics⁎.

Variables	Physical exercise			Vegetable consumption			Fruit consumption			Sleep quality		
	*b*	*SE*	*p*	*b*	*SE*	*p*	*b*	*SE*	*p*	*b*	*SE*	*p*
Network Dynamics												
Friend Rate period 1	7.573	0.362	<0.001	7.713	0.488	<0.001	7.658	0.389	<0.001	7.701	0.432	<0.001
Friend Rate period 2	5.421	0.532	<0.001	5.642	0.698	<0.001	5.451	0.765	<0.001	5.717	0.311	<0.001
Friend Rate period 3	3.562	0.423	<0.001	3.234	0.452	<0.001	3.442	0.652	<0.001	3.743	0.324	<0.001
Outdegree	-3.719	0.054	<0.001	-3.692	0.055	<0.001	-3.718	0.057	<0.001	-3.695	0.051	<0.001
Reciprocity	3.043	0.106	<0.001	3.025	0.093	<0.001	3.048	0.105	<0.001	3.040	0.101	<0.001
Behaviors alter	0.125	0.061	0.043	-0.013	0.027	0.636	0.042	0.029	0.149	0.018	0.086	0.830
Behaviors ego	0.067	0.062	0.279	0.003	0.029	0.909	-0.037	0.027	0.170	-0.143	0.082	0.082
Behaviors similarity	0.203	0.144	0.158	0.011	0.018	0.535	0.121	0.112	0.280	0.508	0.271	0.061
Behavior Dynamics												
Behaviors rate period 1	1.703	0.173	<0.001	3.042	0.415	<0.001	2.488	0.755	<0.001	0.797	0.104	<0.001
Behaviors rate period 2	2.652	0.242	<0.001	4.879	0.341	<0.001	5.142	0.952	<0.001	1.021	0.144	<0.001
Behaviors rate period 3	3.948	0.745	<0.001	5.675	0.356	<0.001	6.345	0.653	<0.001	1.562	0.213	<0.001
Behaviors linear shape	-0.499	0.240	0.038	-0.063	0.114	0.582	-0.007	0.089	0.941	0.338	0.565	0.550
Behaviors quadratic shape	-0.334	0.082	<0.001	-0.054	0.013	<0.001	-0.066	0.009	<0.001	-0.859	0.288	0.002
Behaviors indegree	0.136	0.055	0.014	0.058	0.033	0.082	0.047	0.023	0.039	-0.274	0.192	0.155
Behaviors outdegree	-0.097	0.065	0.136	-0.025	0.037	0.488	-0.033	0.025	0.187	0.274	0.220	0.214
Behaviors average alter	1.591	0.135	<0.001	0.196	0.119	0.100	1.467	0.136	<0.001	1.143	0.160	<0.001

Variables	Smoking			Drinking			Consumption of sugar sweetened beverages			Video game playing		
	b	SE	p	b	SE	p	b	SE	p	b	SE	p
Network Dynamics												
Friend Rate period 1	7.616	0.499	<0.001	7.612	0.385	<0.001	7.515	0.343	<0.001	7.706	0.449	<0.001
Friend Rate period 2	5.152	0.266	<0.001	5.917	0.655	<0.001	5.167	0.423	<0.001	5.109	0.322	<0.001
Friend Rate period 3	3.425	0.623	<0.001	3.452	0.524	<0.001	3.522	0.234	<0.001	3.422	0.421	<0.001
Outdegree	-3.719	0.068	<0.001	-3.704	0.053	<0.001	-3.739	0.064	<0.001	-3.696	0.050	<0.001
Reciprocity	3.040	0.121	<0.001	3.044	0.098	<0.001	3.044	0.105	<0.001	3.027	0.100	<0.001
Behaviors alter	0.239	0.129	0.064	0.037	0.097	0.699	-0.010	0.029	0.736	0.024	0.033	0.471
Behaviors ego	0.206	0.145	0.155	0.229	0.121	0.059	-0.002	0.031	0.956	-0.025	0.034	0.469
Behaviors similarity	0.669	0.524	0.202	0.595	0.476	0.211	1.352	0.403	<0.001	0.519	0.410	0.205
Behavior Dynamics												
Behaviors rate period 1	5.326	1.426	<0.001	1.949	0.252	<0.001	5.804	0.461	<0.001	3.025	0.647	<0.001
Behaviors rate period 2	4.367	1.164	<0.001	1.542	0.376	<0.001	4.397	0.765	<0.001	3.898	0.871	<0.001
Behaviors rate period 3	4.653	1.452	<0.001	2.023	0.523	<0.001	4.652	0.424	<0.001	3.987	0.764	<0.001
Behaviors linear shape	-10.157	7.441	0.172	-0.965	0.255	<0.001	-0.082	0.103	0.429	-0.147	0.169	0.385
Behaviors quadratic shape	0.723	0.308	0.019	0.100	0.060	0.097	-0.099	0.010	<0.001	0.247	0.028	<0.001
Behaviors indegree	0.170	0.133	0.203	0.040	0.051	0.431	-0.014	0.020	0.505	-0.012	0.030	0.694
Behaviors outdegree	0.330	0.509	0.517	-0.013	0.066	0.843	0.036	0.027	0.179	-0.063	0.042	0.132
Behaviors average alter	1.851	0.624	0.003	2.232	0.183	<0.001	0.433	0.226	0.055	2.356	0.103	<0.001

⁎ The full version of SAOM results including control variables can be found in Table S3 in Supplemental Materials.

### Relative contribution of mechanisms

Fig. 2 showed the different mechanisms by which the observed homophily was assigned to generate friend similarity. The findings indicate that, among health-promoting behaviors, contagion effects played a major role in driving homophily for physical activity (49.1%), fruit consumption (44.3%), and sleep (31.4%), while selection effects contributed 5.9%, 3.5%, and 14.7%, respectively. For health-risk behaviors, contagion effects were the dominant contributors to homophily in smoking (56.8%), drinking (64.7%), and online gaming (63.2%). However, homophily in sugar-sweetened beverage consumption was mainly driven by selection effects, which accounted for 39.1% of the similarity.

## Discussion

Using four-wave sociocentric network data, this study disentangled selection and contagion processes underlying homophily across multiple health-related behaviors among university students. Across most domains, including physical activity, fruit consumption, sleep quality, smoking, drinking, and video gaming, contagion effects predominate over selection effects. In contrast, homophily in sugar-sweetened beverage consumption was primarily driven by selective friendship formation. These findings suggest that behavioral similarity within peer networks is not generated by a single universal mechanism. Instead, the relative dominance of contagion and selection varies systematically across behavior types.

Across most behaviors examined in this study, contagion emerged as the dominant mechanism generating homophily. For substance use behaviors such as drinking and smoking, this finding is consistent with prior studies (Ali & Dwyer, 2009; Radó et al., 2024). Importantly, our results extend this pattern to additional behaviors that have been less frequently examined using SAOM, including physical activity, gaming, and sleep. A common feature of these behaviors is that they are socially embedded and often co-performed within university settings. They take place in shared spaces, dormitories, sports facilities, classrooms, and social gatherings, and are highly visible to peers, creating repeated opportunities for behavioral alignment through observation, reinforcement, and normative pressure. During the early university years, when peer acceptance and social integration are particularly salient, students may adjust their behaviors to maintain belonging within newly formed networks (Jusri & Lechner, 2025). Collectively, these findings suggest that contagion processes are especially pronounced for behaviors that are socially coordinated, identity-relevant, and enacted within collective contexts.

A notable exception concerns dietary behaviors: fruit consumption appeared to be primarily shaped by contagion processes, whereas sugar-sweetened beverage consumption was better explained by selection processes. Although both behaviors occur within the same campus environment, they differ in their social organization. Fruit consumption is typically embedded within shared dining settings, where food choices are made simultaneously and in the presence of peers, facilitating visible comparison and potential behavioral alignment (Herman, 2015). In contrast, SSB consumption may be more individualized and temporally dispersed, occurring during solitary study periods or leisure activities, and may reflect more stable personal preferences established prior to university entry (Rosinger et al., 2017). These differences indicate that even within a common residential context, the degree of social coordination and visibility associated with specific behaviors may determine whether similarity arises through peer-induced change or through selective friendship formation (Smith & Christakis, 2008).

Our findings can be situated against two recent stochastic actor-based studies of the same Taiwanese adolescent cohort. Lee et al. (2022) found that similarity in weight status and lifestyle was generated predominantly by homophilous selection, with no peer influence on weight-related outcomes, consistent with our observation that similarity in sugar-sweetened beverage consumption was primarily selection-driven; and Lee et al. (2023) found that adolescents selected friends by class and gender rather than externalizing behavior, while peer substance use predicted individual delinquent behavior, echoing our finding that similarity in smoking and drinking reflected influence rather than selection. Our study diverges, however, in detecting significant contagion across a broader set of behaviors, including health-promoting ones such as fruit consumption and physical activity. One likely explanation lies in developmental stage and network context: the Taiwanese cohort comprised early adolescents (∼12 years) nested within relatively fixed secondary-school classes, whereas our participants were emerging adults (∼18.7 years) forming new peer ties under substantially greater autonomy, during which deliberate friendship formation and intensified co-activity may amplify behavioral contagion.

In terms of contagion strength, health-risk behaviors exhibited stronger contagion effects than health-promoting behaviors, likely due to their addictive nature. Behaviors such as smoking, drinking, and online gaming often involve physiological dependence and psychological reinforcement. For instance, nicotine and alcohol directly stimulate the brain's reward system, enhancing pleasure and dependence, while online gaming reinforces usage through instant feedback and social interaction mechanisms (Bhagat et al., 2020; Koob & Le Moal, 2001). These features accelerate habit formation and enhance peer imitation and contagion within social networks. In contrast, the contagion effects of health-promoting behaviors are generally weaker, possibly because their benefits are long-term, not immediately observable, and lack physiological reinforcement (Glanz et al., 2008). Activities like physical exercise, healthy eating, and regular sleep require sustained effort and resources, with delayed gratification. Even when positively influenced by peers, individuals may struggle to maintain these behaviors due to environmental constraints, low motivation, or limited self-control. This “high cost–low immediate reward” dynamic limits the spontaneous spread of health-promoting behaviors in social networks. From a population health perspective, this imbalance is concerning behaviors with greater long-term harm may diffuse more readily than those that confer long-term benefit. Nevertheless, although the stronger contagion effects observed for certain health-risk behaviors are consistent with addictive mechanisms, this interpretation remains speculative given the absence of biological or longitudinal measures of addiction.

Distinguishing between contagion-dominated and selection-dominated behaviors has direct implications for intervention design. For behaviors primarily shaped by contagion, such as drinking, smoking, gaming, physical activity, and sleep patterns, network-based strategies may be particularly effective (Valente, 2012). Interventions could leverage central actors, reshape perceived norms, or target influential clusters to initiate cascading behavioral change through diffusion processes (Valente, 2012). In such cases, altering the behavior of strategically positioned individuals could, if tested in future intervention studies, generate broader population-level effects. However, despite the theoretical promise of network-based approaches, relatively few studies have applied such interventions to adolescent health-related behaviors (Campbell et al., 2008), particularly beyond substance use contexts. In contrast, for behaviors primarily driven by selection, such as SSB consumption, interventions that focus solely on peer diffusion may have limited impact. Instead, structural or environmental approaches may be more appropriate. Thus, identifying the dominant mechanism underlying homophily is not merely a theoretical exercise. It may help inform whether altering social diffusion pathways or addressing upstream individual and environmental determinants.

This study has several notable strengths, including a high baseline response rate (95.3%) and strong retention across waves (90.3%), which enhances the internal validity of the longitudinal analyses. Nevertheless, several limitations should be considered. First, the use of a purposive sample drawn from two universities may limit the generalizability of the findings to broader populations of young adults. In addition, certain behaviors, particularly smoking, had relatively low prevalence, which may have reduced statistical precision in estimating network effects. The within-cohort boundary captures the dominant peer environment for first-year students in this context but not ties with other grades, pre-university friends, or online contacts; contagion through those channels lies beyond the observed networks. Second, health-related behaviors were assessed through self-report measures, which may be subject to recall error and social desirability bias. Third, friendship ties were modeled as binary, so the intimacy gradient of nominations was not incorporated; because stronger ties may transmit peer influence more strongly than weaker ones, the binary specification may underestimate heterogeneity in contagion across tie strength. Fourth, Analyses were restricted to participants with complete data across all waves; although attrition was low (9.7%), selection bias arising from attrition cannot be fully excluded. Finally, despite the overall high response rate, some demographic subgroups, such as male students, were underrepresented, which may affect the representativeness of specific behavioral estimates.

## Conclusion

In this longitudinal sociocentric study, contagion emerged as the dominant mechanism generating homophily for most socially embedded health behaviors, whereas selection processes predominated for sugar-sweetened beverage consumption. These findings highlight that behavioral similarity within peer networks arises from heterogeneous mechanisms. Recognizing which behaviors are contagion-sensitive versus selection-driven may help inform the design of mechanism-informed public health interventions, which future controlled studies would need to evaluate during the critical transition to adulthood.

## Funding

Not applicable.

## Data availability

The datasets generated and analysed during the current study are not publicly available due to protect study participant privacy but are available from the corresponding author on reasonable request.

## CRediT authorship contribution statement

**Rui Luo:** Conceptualization, Methodology, Formal analysis, Writing – original draft. **Yongqiang Li:** Investigation, Data curation. **Jingyu Zhang:** Investigation, Writing – original draft. **Jinming Liu:** Investigation, Writing – review & editing. **Chun Hao:** Methodology, Writing – review & editing. **Jinghua Li:** Methodology, Writing – review & editing. **Jing Gu:** Conceptualization, Supervision, Project administration, Writing – review & editing.

## Declaration of competing interest

The authors declare that they have no known competing financial interests or personal relationships that could have appeared to influence the work reported in this paper.
