# Supplementary material for: The co-evolution of social networks and health-related behaviors in university students: A two-year longitudinal network study
Source: Int J Clin Health Psychol. 2026 Sep 9;26(3):100720. doi: 10.1016/j.ijchp.2026.100720 (PMC13579669; doi:10.1016/j.ijchp.2026.100720)
Supplement: Supplementary file 1 [file mmc1.docx]

Table S1. Description and graphical representation of the included model parameters.

| Model parameter | Description |
| --- | --- |
| Network dynamics |  |
| Out-degree | Overall tendency to nominate friends. |
| Reciprocity | The tendency to reciprocate friendship nomination. |
| Transitive triplets | The tendency for friends of a friend to become friends. |
| 3-cycles | In a network, three people are connected to each other to form a closed triplet. |
| Covariates alter | Certain covariate properties make individuals more likely to be nominated as friends by other peers. |
| Covariates ego | Certain covariate properties make individuals more likely to nominate others as friends. |
| Covariates similarity | The tendency to select friends based on similar covariate attributes. |
| Behaviors alter | There is a tendency where individuals engaging in certain health-related behaviors are more likely to be nominated as friends by their peers compared to those who do not engage in such health-related behaviors. |
| Behaviors ego | There is a tendency where engaging in certain health-related behaviors increases the likelihood of being nominated as a friend compared to those who do not engage in such behaviors. |
| Behaviors similarity | The tendency to choose friends based on similar health-related behaviors |
| Behavior Dynamics |  |
| Control variables |  |
| Behaviors linear shape | Linear component in basic preference function for health-related behaviors |
| Behaviors quadratic shape | Squared component in basic preference function for health-related behaviors. |
| Behaviors indegree | The influence of one's own popularity in the network on health-related behaviors. |
| Behaviors outdegree | The effect of one's own activity in the network on one's health-related behaviors. |
| Behaviors: effect from Covariates | Effects of covariates on health-related behaviors. |
| Behaviors average alter | The tendency for adolescents to adjust their health-related behaviors to align with those of their friends, either becoming similar. |

Table S2. Coding of health-related behaviors for the SAOM behavioral submodel

| **Behavior** | **Final SAOM coding (per GOF)** | **SAOM category values (as modeled)** | **Categories collapsed** | **Reverse-coded** | **Higher value indicates** |
| --- | --- | --- | --- | --- | --- |
| Physical exercise | 4 categories (0–3) | 0 = never; 1 = low (≤19); 2 = moderate (20–42); 3 = high (≥43) | Yes | No | More physical activity |
| Fruit consumption | 9 categories (0–8) | 0 = never; 1 = 1/month; 2 = 2–3/month; 3 = 1/week; 4 = 2/week; 5 = 3–4/week; 6 = 5–6/week; 7 = 1/day; 8 = ≥2/day | No | No | Higher fruit intake |
| Vegetable consumption | 9 categories (0–8) | 0 = never; 1 = 1/month; 2 = 2–3/month; 3 = 1/week; 4 = 2/week; 5 = 3–4/week; 6 = 5–6/week; 7 = 1/day; 8 = ≥2/day | No | No | Higher vegetable intake |
| Sleep quality | 2 categories (0–1) | 0 = poor (PSQI >5); 1 = good (PSQI ≤5) | Yes | Yes | Better sleep quality |
| Smoking | 7 categories (0–6) | 0 = never; 1 = 1–2 days; 2 = 3–5 days; 3 = 6–9 days; 4 = 10–19 days; 5 = 20–29 days; 6 = every day | No | No | More smoking |
| Drinking | 5 categories (0–4) | 0 = never; 1 = ≤once/month; 2 = 2–4/month; 3 = 2–3/week; 4 = ≥4/week | No | No | More frequent drinking |
| Sugar-sweetened beverage (SSB) consumption | 7 categories (0–6) | 0 = never; 1 = <once/week; 2 = 1–2/week; 3 = 3–4/week; 4 = 5–6/week; 5 = once/day; 6 = ≥2/day | Yes | No | Higher SSB consumption |
| Video game playing | 5 categories (0–4) | 0 = never; 1 = ≤3 h/week; 2 = 3–7 h/week; 3 = 7–14 h/week; 4 = >14 h/week | Yes | No | More gaming time |

*Note. Final SAOM coding gives the number of ordered categories in the fitted behavior variables (goodness-of-fit behavior distributions, School 1). Fruit and vegetable consumption were each a single FFQ item on a 9-point frequency scale (0–8). Smoking used the full seven-category past-month scale; Table 3 presents a collapsed five-category grouping for display. Sleep was reverse-coded so higher = better sleep. For drinking, the School-1 GOF shows four categories because the highest (≥4/week) was unobserved there.*

Table S3. SAOM estimates for friendship network and health-related behaviors dynamics (full version)

| Variables | Physical exercise | | |  | Vegetable consumption | | |  | Fruit consumption | | |  | Sleep quality | | |
| --- | --- | --- | --- | --- | --- | --- | --- | --- | --- | --- | --- | --- | --- | --- | --- |
|  | *b* | *SE* | *p* |  | *b* | *SE* | *p* |  | *b* | *SE* | *p* |  | *b* | *SE* | *p* |
| Network Dynamics |  |  |  |  |  |  |  |  |  |  |  |  |  |  |  |
| 1. friend Rate period 1 | 7.573 | 0.362 | <0.001 |  | 7.713 | 0.488 | <0.001 |  | 7.658 | 0.389 | <0.001 |  | 7.701 | 0.432 | <0.001 |
| 2. friend Rate period 2 | 5.421 | 0.532 | <0.001 |  | 5.642 | 0.698 | <0.001 |  | 5.451 | 0.765 | <0.001 |  | 5.717 | 0.311 | <0.001 |
| 3. friend Rate period 3 | 3.562 | 0.423 | <0.001 |  | 3.234 | 0.452 | <0.001 |  | 3.442 | 0.652 | <0.001 |  | 3.743 | 0.324 | <0.001 |
| 4. outdegree | -3.719 | 0.054 | <0.001 |  | -3.692 | 0.055 | <0.001 |  | -3.718 | 0.057 | <0.001 |  | -3.695 | 0.051 | <0.001 |
| 5. reciprocity | 3.043 | 0.106 | <0.001 |  | 3.025 | 0.093 | <0.001 |  | 3.048 | 0.105 | <0.001 |  | 3.040 | 0.101 | <0.001 |
| 6. transitive triplets | 0.745 | 0.037 | <0.001 |  | 0.727 | 0.035 | <0.001 |  | 0.732 | 0.037 | <0.001 |  | 0.728 | 0.035 | <0.001 |
| 7. 3-cycles | -0.604 | 0.082 | <0.001 |  | -0.587 | 0.070 | <0.001 |  | -0.589 | 0.084 | <0.001 |  | -0.594 | 0.072 | <0.001 |
| 8. gender alter | -0.144 | 0.097 | 0.140 |  | -0.087 | 0.095 | 0.359 |  | -0.120 | 0.085 | 0.156 |  | -0.079 | 0.091 | 0.386 |
| 9. gender ego | 0.005 | 0.101 | 0.961 |  | 0.037 | 0.098 | 0.705 |  | 0.044 | 0.090 | 0.624 |  | 0.052 | 0.104 | 0.619 |
| 10. gender similarity | 0.739 | 0.098 | <0.001 |  | 0.738 | 0.087 | <0.001 |  | 0.745 | 0.089 | <0.001 |  | 0.743 | 0.080 | <0.001 |
| 11. only-child status alter | -0.061 | 0.078 | 0.429 |  | -0.061 | 0.073 | 0.408 |  | -0.063 | 0.078 | 0.424 |  | -0.060 | 0.080 | 0.448 |
| 12. only-child status ego | 0.200 | 0.080 | 0.013 |  | 0.199 | 0.080 | 0.013 |  | 0.202 | 0.090 | 0.025 |  | 0.206 | 0.077 | 0.008 |
| 13. only-child status similarity | -0.126 | 0.071 | 0.079 |  | -0.122 | 0.065 | 0.062 |  | -0.125 | 0.081 | 0.122 |  | -0.124 | 0.067 | 0.065 |
| 14. parental education alter | -0.112 | 0.039 | 0.004 |  | -0.115 | 0.039 | 0.004 |  | -0.117 | 0.036 | 0.001 |  | -0.116 | 0.036 | 0.001 |
| 15. parental education ego | -0.003 | 0.039 | 0.943 |  | -0.004 | 0.038 | 0.916 |  | -0.008 | 0.043 | 0.860 |  | -0.009 | 0.038 | 0.805 |
| 16. parental education similarity | 0.521 | 0.133 | <0.001 |  | 0.534 | 0.133 | <0.001 |  | 0.536 | 0.133 | <0.001 |  | 0.544 | 0.131 | <0.001 |
| 17. household registration alter | 0.023 | 0.081 | 0.775 |  | 0.030 | 0.077 | 0.699 |  | 0.041 | 0.082 | 0.618 |  | 0.032 | 0.077 | 0.677 |
| 18. household registration ego | -0.005 | 0.082 | 0.948 |  | 0.003 | 0.085 | 0.973 |  | -0.001 | 0.082 | 0.991 |  | -0.002 | 0.078 | 0.980 |
| 19. household registration similarity | 0.092 | 0.070 | 0.194 |  | 0.088 | 0.064 | 0.172 |  | 0.087 | 0.070 | 0.215 |  | 0.088 | 0.061 | 0.153 |
| 20. monthly income alter | 0.024 | 0.046 | 0.598 |  | 0.005 | 0.042 | 0.904 |  | -0.007 | 0.046 | 0.886 |  | 0.001 | 0.040 | 0.982 |
| 21. monthly income ego | 0.091 | 0.047 | 0.051 |  | 0.080 | 0.043 | 0.064 |  | 0.091 | 0.046 | 0.049 |  | 0.076 | 0.047 | 0.107 |
| 22. monthly income similarity | 0.415 | 0.207 | 0.045 |  | 0.388 | 0.201 | 0.054 |  | 0.419 | 0.229 | 0.068 |  | 0.367 | 0.198 | 0.065 |
| 23. BMI alter | 0.034 | 0.037 | 0.357 |  | 0.034 | 0.035 | 0.326 |  | 0.031 | 0.033 | 0.362 |  | 0.036 | 0.033 | 0.283 |
| 24. BMI ego | 0.030 | 0.039 | 0.451 |  | 0.033 | 0.033 | 0.318 |  | 0.036 | 0.037 | 0.335 |  | 0.033 | 0.038 | 0.387 |
| 25. BMI similarity | -0.063 | 0.105 | 0.546 |  | -0.079 | 0.100 | 0.431 |  | -0.068 | 0.112 | 0.544 |  | -0.071 | 0.101 | 0.485 |
| 26. behaviors alter | 0.125 | 0.061 | 0.043 |  | -0.013 | 0.027 | 0.636 |  | 0.042 | 0.029 | 0.149 |  | 0.018 | 0.086 | 0.830 |

(Continued) Table S3. SAOM estimates for friendship network and health-related behaviors dynamics (full version)

| Variables | Physical exercise | | |  | Vegetable consumption | | |  | Fruit consumption | | |  | Sleep quality | | |
| --- | --- | --- | --- | --- | --- | --- | --- | --- | --- | --- | --- | --- | --- | --- | --- |
|  | *b* | *SE* | *p* |  | *b* | *SE* | *p* |  | *b* | *SE* | *p* |  | *b* | *SE* | *p* |
| 27. behaviors ego | 0.067 | 0.062 | 0.279 |  | 0.003 | 0.029 | 0.909 |  | -0.037 | 0.027 | 0.170 |  | -0.143 | 0.082 | 0.082 |
| 28. behaviors similarity | 0.203 | 0.144 | 0.158 |  | 0.011 | 0.018 | 0.535 |  | 0.121 | **0.112** | 0.280 |  | 0.508 | 0.271 | 0.061 |
| Behavior Dynamics |  |  |  |  |  |  |  |  |  |  |  |  |  |  |  |
| 29. behaviors rate period 1 | 1.703 | 0.173 | <0.001 |  | 3.042 | 0.415 | <0.001 |  | 2.488 | 0.755 | <0.001 |  | 0.797 | 0.104 | <0.001 |
| 30. behaviors rate period 2 | 2.652 | 0.242 | <0.001 |  | 4.879 | 0.341 | <0.001 |  | 5.142 | 0.952 | <0.001 |  | 1.021 | 0.144 | <0.001 |
| 31. behaviors rate period 3 | 3.948 | 0.745 | <0.001 |  | 5.675 | 0.356 | <0.001 |  | 6.345 | 0.653 | <0.001 |  | 1.562 | 0.213 | <0.001 |
| 32. behaviors linear shape | -0.499 | 0.240 | 0.038 |  | -0.063 | 0.114 | 0.582 |  | -0.007 | 0.089 | 0.941 |  | 0.338 | 0.565 | 0.550 |
| 33. behaviors quadratic shape | -0.334 | 0.082 | <0.001 |  | -0.054 | 0.013 | <0.001 |  | -0.066 | 0.009 | <0.001 |  | -0.859 | 0.288 | 0.002 |
| 34. behaviors indegree | 0.136 | 0.055 | 0.014 |  | 0.058 | 0.033 | 0.082 |  | 0.047 | 0.023 | 0.039 |  | -0.274 | 0.192 | 0.155 |
| 35. behaviors outdegree | -0.097 | 0.065 | 0.136 |  | -0.025 | 0.037 | 0.488 |  | -0.033 | 0.025 | 0.187 |  | 0.274 | 0.220 | 0.214 |
| 36. behaviors average alter | 1.591 | 0.135 | <0.001 |  | 0.196 | **0.119** | 0.100 |  | 1.467 | 0.136 | <0.001 |  | 1.143 | 0.160 | <0.001 |
| 37. behaviors: effect from gender | -0.041 | 0.168 | 0.806 |  | 0.046 | 0.069 | 0.501 |  | 0.012 | 0.056 | 0.835 |  | 0.213 | 0.329 | 0.518 |
| 38. behaviors: effect from only-child status | 0.119 | 0.165 | 0.472 |  | 0.068 | 0.081 | 0.401 |  | 0.032 | 0.063 | 0.612 |  | 0.692 | 0.374 | 0.065 |
| 39. behaviors: effect from parental education | -0.019 | 0.077 | 0.810 |  | 0.029 | 0.039 | 0.455 |  | 0.016 | 0.035 | 0.651 |  | 0.161 | 0.178 | 0.366 |
| 40. behaviors: effect from household | 0.172 | 0.163 | 0.291 |  | -0.027 | 0.080 | 0.736 |  | 0.111 | 0.068 | 0.101 |  | -0.180 | 0.359 | 0.616 |
| 41. behaviors: effect from monthly income | 0.187 | 0.091 | 0.041 |  | 0.011 | 0.044 | 0.800 |  | 0.018 | 0.036 | 0.612 |  | -0.245 | 0.214 | 0.254 |
| 42. behaviors: effect from BMI | 0.058 | 0.067 | 0.386 |  | 0.003 | 0.034 | 0.921 |  | 0.028 | 0.028 | 0.323 |  | 0.104 | 0.173 | 0.549 |

、

(Continued) Table S3. SAOM estimates for friendship network and health-related behaviors dynamics (full version)

| Variables | Smoking | | |  | Drinking | | |  | Consumption of sugar sweetened beverages | | |  | Video game playing | | |
| --- | --- | --- | --- | --- | --- | --- | --- | --- | --- | --- | --- | --- | --- | --- | --- |
|  | *b* | *SE* | *p* |  | *b* | *SE* | *p* |  | *b* | *SE* | *p* |  | *b* | *SE* | *p* |
| Network Dynamics |  |  |  |  |  |  |  |  |  |  |  |  |  |  |  |
| 1. friend Rate period 1 | 7.616 | 0.499 | <0.001 |  | 7.612 | 0.385 | <0.001 |  | 7.515 | 0.343 | <0.001 |  | 7.706 | 0.449 | <0.001 |
| 2. friend Rate period 2 | 5.152 | 0.266 | <0.001 |  | 5.917 | 0.655 | <0.001 |  | 5.167 | 0.423 | <0.001 |  | 5.109 | 0.322 | <0.001 |
| 3. friend Rate period 3 | 3.425 | 0.623 | <0.001 |  | 3.452 | 0.524 | <0.001 |  | 3.522 | 0.234 | <0.001 |  | 3.422 | 0.421 | <0.001 |
| 4. outdegree | -3.719 | 0.068 | <0.001 |  | -3.704 | 0.053 | <0.001 |  | -3.739 | 0.064 | <0.001 |  | -3.696 | 0.050 | <0.001 |
| 5. reciprocity | 3.040 | 0.121 | <0.001 |  | 3.044 | 0.098 | <0.001 |  | 3.044 | 0.105 | <0.001 |  | 3.027 | 0.100 | <0.001 |
| 6. transitive triplets | 0.731 | 0.041 | <0.001 |  | 0.734 | 0.037 | <0.001 |  | 0.737 | 0.038 | <0.001 |  | 0.726 | 0.034 | <0.001 |
| 7. 3-cycles | -0.591 | 0.093 | <0.001 |  | -0.599 | 0.069 | <0.001 |  | -0.591 | 0.079 | <0.001 |  | -0.587 | 0.079 | <0.001 |
| 8. gender alter | -0.067 | 0.105 | 0.526 |  | -0.096 | 0.092 | 0.296 |  | -0.084 | 0.091 | 0.351 |  | -0.063 | 0.099 | 0.525 |
| 9. gender ego | 0.056 | 0.097 | 0.563 |  | 0.053 | 0.091 | 0.557 |  | 0.034 | 0.091 | 0.708 |  | 0.004 | 0.104 | 0.965 |
| 10. gender similarity | 0.769 | 0.085 | <0.001 |  | 0.752 | 0.082 | <0.001 |  | 0.743 | 0.090 | <0.001 |  | 0.733 | 0.090 | <0.001 |
| 11. only-child status alter | -0.070 | 0.086 | 0.413 |  | -0.069 | 0.071 | 0.332 |  | -0.059 | 0.076 | 0.435 |  | -0.061 | 0.081 | 0.448 |
| 12. only-child status ego | 0.200 | 0.105 | 0.057 |  | 0.214 | 0.082 | 0.009 |  | 0.208 | 0.078 | 0.008 |  | 0.201 | 0.090 | 0.027 |
| 13. only-child status similarity | -0.134 | 0.079 | 0.092 |  | -0.129 | 0.071 | 0.070 |  | -0.121 | 0.070 | 0.082 |  | -0.126 | 0.073 | 0.086 |
| 14. parental education alter | -0.120 | 0.044 | 0.007 |  | -0.114 | 0.036 | 0.002 |  | -0.113 | 0.037 | 0.002 |  | -0.115 | 0.036 | 0.001 |
| 15. parental education ego | -0.004 | 0.043 | 0.922 |  | -0.006 | 0.035 | 0.858 |  | 0.000 | 0.041 | 0.998 |  | -0.002 | 0.041 | 0.943 |
| 16. parental education similarity | 0.558 | 0.255 | 0.029 |  | 0.540 | 0.152 | 0.001 |  | 0.526 | 0.140 | <0.001 |  | 0.533 | 0.140 | <0.001 |
| 17. household registration alter | 0.026 | 0.077 | 0.730 |  | 0.024 | 0.084 | 0.775 |  | 0.028 | 0.076 | 0.714 |  | 0.033 | 0.073 | 0.652 |
| 18. household registration ego | 0.008 | 0.102 | 0.934 |  | 0.003 | 0.076 | 0.968 |  | 0.003 | 0.087 | 0.972 |  | 0.002 | 0.088 | 0.978 |
| 19. household registration similarity | 0.071 | 0.072 | 0.326 |  | 0.087 | 0.061 | 0.157 |  | 0.089 | 0.065 | 0.177 |  | 0.089 | 0.069 | 0.198 |
| 20. monthly income alter | 0 | 0.048 | 1 |  | 0.008 | 0.043 | 0.839 |  | 0.006 | 0.046 | 0.904 |  | 0.003 | 0.042 | 0.941 |
| 21. monthly income ego | 0.095 | 0.058 | 0.101 |  | 0.067 | 0.043 | 0.120 |  | 0.080 | 0.046 | 0.081 |  | 0.078 | 0.044 | 0.079 |
| 22. monthly income similarity | 0.391 | 0.194 | 0.045 |  | 0.364 | 0.213 | 0.087 |  | 0.457 | 0.213 | 0.032 |  | 0.381 | 0.222 | 0.087 |
| 23. BMI alter | 0.041 | 0.047 | 0.381 |  | 0.030 | 0.032 | 0.343 |  | 0.032 | 0.036 | 0.376 |  | 0.035 | 0.032 | 0.274 |
| 24. BMI ego | 0.040 | 0.038 | 0.295 |  | 0.041 | 0.035 | 0.246 |  | 0.026 | 0.035 | 0.458 |  | 0.031 | 0.036 | 0.386 |
| 25. BMI similarity | -0.044 | 0.152 | 0.770 |  | -0.074 | 0.110 | 0.498 |  | -0.069 | 0.106 | 0.518 |  | -0.084 | 0.114 | 0.458 |

(Continued) Table S3. SAOM estimates for friendship network and health-related behaviors dynamics (full version)

| Variables | Smoking | | |  | Drinking | | |  | Consumption of sugar sweetened beverages | | |  | Video game playing | | |
| --- | --- | --- | --- | --- | --- | --- | --- | --- | --- | --- | --- | --- | --- | --- | --- |
|  | *b* | *SE* | *p* |  | *b* | *SE* | *p* |  | *b* | *SE* | *p* |  | *b* | *SE* | *p* |
| 26. behaviors alter | 0.239 | **0.129** | 0.064 |  | 0.037 | 0.097 | 0.699 |  | -0.010 | 0.029 | 0.736 |  | 0.024 | 0.033 | 0.471 |
| 27. behaviors ego | 0.206 | 0.145 | 0.155 |  | 0.229 | 0.121 | 0.059 |  | -0.002 | 0.031 | 0.956 |  | -0.025 | 0.034 | 0.469 |
| 28. behaviors similarity | 0.669 | 0.524 | 0.202 |  | 0.595 | **0.476** | 0.211 |  | 1.352 | 0.403 | <0.001 |  | 0.519 | 0.410 | 0.205 |
| Behavior Dynamics |  |  |  |  |  |  |  |  |  |  |  |  |  |  |  |
| 29. behaviors rate period 1 | 5.326 | 1.426 | <0.001 |  | 1.949 | 0.252 | <0.001 |  | 5.804 | 0.461 | <0.001 |  | 3.025 | 0.647 | <0.001 |
| 30. behaviors rate period 2 | 4.367 | 1.164 | <0.001 |  | 1.542 | 0.376 | <0.001 |  | 4.397 | 0.765 | <0.001 |  | 3.898 | 0.871 | <0.001 |
| 31. behaviors rate period 3 | 4.653 | 1.452 | <0.001 |  | 2.023 | 0.523 | <0.001 |  | 4.652 | 0.424 | <0.001 |  | 3.987 | 0.764 | <0.001 |
| 32. behaviors linear shape | -10.157 | 7.441 | 0.172 |  | -0.965 | 0.255 | <0.001 |  | -0.082 | 0.103 | 0.429 |  | -0.147 | 0.169 | 0.385 |
| 33. behaviors quadratic shape | 0.723 | 0.308 | 0.019 |  | 0.100 | 0.060 | 0.097 |  | -0.099 | 0.010 | <0.001 |  | 0.247 | 0.028 | <0.001 |
| 34. behaviors indegree | 0.170 | 0.133 | 0.203 |  | 0.040 | 0.051 | 0.431 |  | -0.014 | 0.020 | 0.505 |  | -0.012 | 0.030 | 0.694 |
| 35. behaviors outdegree | 0.330 | 0.509 | 0.517 |  | -0.013 | 0.066 | 0.843 |  | 0.036 | 0.027 | 0.179 |  | -0.063 | 0.042 | 0.132 |
| 36. behaviors average alter | 1.851 | 0.624 | 0.003 |  | 2.232 | 0.183 | <0.001 |  | 0.433 | 0.226 | 0.055 |  | 2.356 | 0.103 | <0.001 |
| 37. behaviors: effect from gender | 0.674 | 1.494 | 0.651 |  | 0.106 | 0.052 | 0.041 |  | 0.032 | 0.061 | 0.603 |  | -0.160 | 0.143 | 0.261 |
| 38. behaviors: effect from only-child status | 1.079 | 3.177 | 0.734 |  | 0.197 | 0.319 | 0.537 |  | 0.061 | 0.065 | 0.349 |  | 0.041 | 0.106 | 0.697 |
| 39. behaviors: effect from parental education | -0.237 | 0.819 | 0.772 |  | 0.099 | 0.091 | 0.278 |  | -0.009 | 0.033 | 0.782 |  | 0.057 | 0.059 | 0.334 |
| 40. behaviors: effect from household | -0.632 | 1.192 | 0.596 |  | 0.134 | 0.127 | 0.292 |  | -0.056 | 0.067 | 0.398 |  | 0.022 | 0.117 | 0.849 |
| 41. behaviors: effect from monthly income | 0.754 | 1.202 | 0.530 |  | 0.085 | 0.123 | 0.491 |  | 0.004 | 0.039 | 0.923 |  | 0.081 | 0.060 | 0.174 |
| 42. behaviors: effect from BMI | −0.661 | 1.001 | 0.509 |  | 0.077 | 0.112 | 0.492 |  | 0.058 | 0.030 | 0.058 |  | 0.886 | 1.199 | 0.460 |

Table S4. Convergence diagnostics of the school-specific stochastic actor-oriented models.

| Behavior | School | Maximum convergence ratio | Maximum \|t\| |
| --- | --- | --- | --- |
| Physical exercise | 1 | 0.1581 | 0.0881 |
| Physical exercise | 2 | 0.1452 | 0.0767 |
| Physical exercise | 3 | 0.1343 | 0.0691 |
| Physical exercise | 4 | 0.1381 | 0.0885 |
| Physical exercise | 5 | 0.1711 | 0.0638 |
| Fruit consumption | 1 | 0.1543 | 0.0881 |
| Fruit consumption | 2 | 0.1289 | 0.0997 |
| Fruit consumption | 3 | 0.1512 | 0.0734 |
| Fruit consumption | 4 | 0.1135 | 0.0815 |
| Fruit consumption | 5 | 0.1571 | 0.0538 |
| Vegetable consumption | 1 | 0.1422 | 0.0819 |
| Vegetable consumption | 2 | 0.1722 | 0.0518 |
| Vegetable consumption | 3 | 0.1522 | 0.0761 |
| Vegetable consumption | 4 | 0.1034 | 0.0425 |
| Vegetable consumption | 5 | 0.1331 | 0.0867 |
| Sleep quality | 1 | 0.1638 | 0.0802 |
| Sleep quality | 2 | 0.1482 | 0.0958 |
| Sleep quality | 3 | 0.1764 | 0.0940 |
| Sleep quality | 4 | 0.1189 | 0.0522 |
| Sleep quality | 5 | 0.1624 | 0.0818 |
| Drinking | 1 | 0.1434 | 0.0611 |
| Drinking | 2 | 0.1644 | 0.0906 |
| Drinking | 3 | 0.1562 | 0.0701 |
| Drinking | 4 | 0.1601 | 0.0611 |
| Drinking | 5 | 0.1425 | 0.0837 |
| Smoking | 1 | 0.1627 | 0.0772 |
| Smoking | 2 | 0.1933 | 0.0791 |
| Smoking | 3 | 0.1772 | 0.0520 |
| Smoking | 4 | 0.1309 | 0.0621 |
| Smoking | 5 | 0.1545 | 0.0762 |
| Consumption of sugar-sweetened beverages | 1 | 0.1365 | 0.0515 |
| Consumption of sugar-sweetened beverages | 2 | 0.1907 | 0.0815 |
| Consumption of sugar-sweetened beverages | 3 | 0.1113 | 0.0755 |
| Consumption of sugar-sweetened beverages | 4 | 0.1533 | 0.0532 |
| Consumption of sugar-sweetened beverages | 5 | 0.1253 | 0.0721 |
| Video game playing | 1 | 0.1902 | 0.0865 |
| Video game playing | 2 | 0.1504 | 0.0829 |
| Video game playing | 3 | 0.1303 | 0.0651 |
| Video game playing | 4 | 0.1447 | 0.0673 |
| Video game playing | 5 | 0.1123 | 0.0760 |

Note. Convergence was assessed by the overall maximum convergence ratio and the maximum absolute t-ratio across parameters. A model was considered converged when the overall maximum convergence ratio was < 0.25 and all parameter t-ratios were < 0.10.

Table S5. Goodness-of-fit *p*-values for each school-specific model.

| Behavior | School | Outdegree | Indegree | Behavior |
| --- | --- | --- | --- | --- |
| Physical exercise | 1 | 0.240 | 0.132 | 0.675 |
| Physical exercise | 2 | 0.575 | 0.423 | 0.549 |
| Physical exercise | 3 | 0.314 | 0.258 | 0.719 |
| Physical exercise | 4 | 0.128 | 0.061 | 0.221 |
| Physical exercise | 5 | 0.840 | 0.088 | 0.063 |
| Fruit consumption | 1 | 0.246 | 0.137 | 0.230 |
| Fruit consumption | 2 | 0.582 | 0.419 | 0.084 |
| Fruit consumption | 3 | 0.320 | 0.264 | 0.068 |
| Fruit consumption | 4 | 0.133 | 0.058 | 0.032 |
| Fruit consumption | 5 | 0.848 | 0.090 | 0.110 |
| Vegetable consumption | 1 | 0.234 | 0.129 | 0.058 |
| Vegetable consumption | 2 | 0.568 | 0.431 | 0.097 |
| Vegetable consumption | 3 | 0.307 | 0.252 | 0.080 |
| Vegetable consumption | 4 | 0.122 | 0.066 | 0.187 |
| Vegetable consumption | 5 | 0.833 | 0.083 | 0.185 |
| Sleep quality | 1 | 0.241 | 0.141 | 0.479 |
| Sleep quality | 2 | 0.579 | 0.425 | 0.408 |
| Sleep quality | 3 | 0.315 | 0.267 | 0.222 |
| Sleep quality | 4 | 0.130 | 0.049 | 0.542 |
| Sleep quality | 5 | 0.844 | 0.095 | 0.168 |
| Drinking | 1 | 0.249 | 0.131 | 0.114 |
| Drinking | 2 | 0.571 | 0.416 | 0.271 |
| Drinking | 3 | 0.309 | 0.249 | 0.042 |
| Drinking | 4 | 0.125 | 0.070 | 0.664 |
| Drinking | 5 | 0.837 | 0.086 | 0.067 |
| Smoking | 1 | 0.236 | 0.124 | 0.153 |
| Smoking | 2 | 0.584 | 0.433 | 0.033 |
| Smoking | 3 | 0.318 | 0.258 | 0.119 |
| Smoking | 4 | 0.136 | 0.061 | 0.155 |
| Smoking | 5 | 0.842 | 0.079 | 0.332 |
| Consumption of sugar-sweetened beverages | 1 | 0.243 | 0.135 | 0.130 |
| Consumption of sugar-sweetened beverages | 2 | 0.577 | 0.421 | 0.336 |
| Consumption of sugar-sweetened beverages | 3 | 0.312 | 0.268 | 0.191 |
| Consumption of sugar-sweetened beverages | 4 | 0.120 | 0.052 | 0.450 |
| Consumption of sugar-sweetened beverages | 5 | 0.835 | 0.098 | 0.261 |
| Video game playing | 1 | 0.238 | 0.142 | 0.391 |
| Video game playing | 2 | 0.569 | 0.427 | 0.162 |
| Video game playing | 3 | 0.323 | 0.255 | 0.648 |
| Video game playing | 4 | 0.129 | 0.068 | 0.420 |
| Video game playing | 5 | 0.846 | 0.088 | 0.074 |

Note. p-values from sienaGOF (1,000 simulations). *p* > 0.05 indicates the simulated distribution is consistent with the observed data. Distributions: outdegree, indegree, behavior distribution. Values reported as < 0.001 were 0 in the empirical Monte Carlo distribution.

Table S6. Time-heterogeneity tests (sienaTimeTest) for each school-specific model.

| Behavior | School | Joint chi-square | p |
| --- | --- | --- | --- |
| Physical exercise | 1 | 86.78 | 0.113 |
| Physical exercise | 2 | 71.34 | 0.500 |
| Physical exercise | 3 | 68.68 | 0.589 |
| Physical exercise | 4 | 72.15 | 0.473 |
| Physical exercise | 5 | 75.88 | 0.355 |
| Fruit consumption | 1 | 83.23 | 0.172 |
| Fruit consumption | 2 | 89.34 | 0.081 |
| Fruit consumption | 3 | 80.20 | 0.238 |
| Fruit consumption | 4 | 52.04 | 0.963 |
| Fruit consumption | 5 | 61.22 | 0.814 |
| Vegetable consumption | 1 | 71.69 | 0.488 |
| Vegetable consumption | 2 | 73.81 | 0.419 |
| Vegetable consumption | 3 | 58.28 | 0.879 |
| Vegetable consumption | 4 | 44.02 | 0.996 |
| Vegetable consumption | 5 | 69.72 | 0.554 |
| Sleep quality | 1 | 78.12 | 0.291 |
| Sleep quality | 2 | 56.79 | 0.905 |
| Sleep quality | 3 | 70.59 | 0.525 |
| Sleep quality | 4 | 56.53 | 0.910 |
| Sleep quality | 5 | 54.89 | 0.933 |
| Smoking | 1 | 59.22 | 0.823 |
| Smoking | 2 | 64.21 | 0.722 |
| Smoking | 3 | 50.09 | 0.969 |
| Smoking | 4 | 78.01 | 0.315 |
| Smoking | 5 | 66.95 | 0.598 |
| Drinking | 1 | 89.02 | 0.085 |
| Drinking | 2 | 87.10 | 0.109 |
| Drinking | 3 | 85.77 | 0.128 |
| Drinking | 4 | 77.04 | 0.321 |
| Drinking | 5 | 63.29 | 0.758 |
| Consumption of sugar-sweetened beverages | 1 | 71.52 | 0.494 |
| Consumption of sugar-sweetened beverages | 2 | 52.90 | 0.956 |
| Consumption of sugar-sweetened beverages | 3 | 51.73 | 0.966 |
| Consumption of sugar-sweetened beverages | 4 | 45.04 | 0.995 |
| Consumption of sugar-sweetened beverages | 5 | 58.76 | 0.869 |
| Video game playing | 1 | 59.40 | 0.856 |
| Video game playing | 2 | 75.78 | 0.358 |
| Video game playing | 3 | 71.91 | 0.481 |
| Video game playing | 4 | 80.37 | 0.234 |
| Video game playing | 5 | 72.30 | 0.468 |

Note. Joint score-type test of time heterogeneity across the three observation periods. *p* > 0.05 indicates no significant time heterogeneity.

Figure S1. Goodness-of-fit for the behavior distributions of the eight health-related behaviors (School 1).

Note: Panels (a)–(h) correspond to physical exercise, fruit consumption, vegetable consumption, sleep quality, drinking, smoking, sugar-sweetened beverage consumption, and video game playing. Observed values (red) are compared with distributions simulated from the fitted stochastic actor-oriented models; *p* > 0.05 indicates adequate fit.

Figure S2. Goodness-of-fit for the indegree distribution of the friendship networks in each of the five schools (panels a–e, Schools 1–5).

Note: Violin/box plots show the statistic in networks simulated from the fitted stochastic actor-oriented models; red points and lines denote the observed values. Monte Carlo p-values (Mahalanobis distance) are shown below each panel; *p* > 0.05 indicates adequate fit.
